# Long-term outcomes of the nano-hydroxyapatite/polyamide-66 cage versus the titanium mesh cage for anterior reconstruction of thoracic and lumbar corpectomy: a retrospective study with at least 7 years of follow-up

**DOI:** 10.1186/s13018-023-03951-x

**Published:** 2023-07-05

**Authors:** Bowen Hu, Liang Wang, Yueming Song, Xi Yang, Limin Liu, Chunguang Zhou

**Affiliations:** Department of Orthopedic Surgery and Orthopedic Research Institute, West China Hospital, Sichuan University, No. 37 GuoXue Road, Chengdu, 610041 Sichuan China

**Keywords:** Traumatic thoracolumbar fracture, Anterior spinal fusion, Long-term follow-up, Nano hydroxyapatite/polyamide 66 (n-HA/PA66) cage, Titanium mesh cage

## Abstract

**Background:**

The nano-hydroxyapatite/polyamide-66 (n-HA/PA66) cage is a biomimetic cage with a lower elastic modulus than the titanium mesh cage (TMC). This study aimed to compare the long-term outcomes of the n-HA/PA66 cage and TMC in the anterior reconstruction of thoracic and lumbar fractures.

**Methods:**

We retrospectively studied 113 patients with acute traumatic thoracic or lumbar burst fractures, comprising 60 patients treated with the TMC and 53 treated with the n-HA/PA66 cage for anterior reconstruction following single-level corpectomy. The radiographic data (cage subsidence, fusion status, segmental sagittal alignment) and clinical data (visual analogue scale (VAS) for pain and Oswestry Disability Index (ODI) for function) were evaluated preoperatively, postoperatively, and at final follow-up after a minimum 7-year period.

**Results:**

The n-HA/PA66 and TMC groups had similar final fusion rates (96.2% vs. 95.0%). The cage subsidence at final follow-up was 2.3 ± 1.6 mm with subsidence of more than 3 mm occurring in 24.5% in the n-HA/PA66 group, which was significantly lower than the respective values of 3.9 ± 2.5 mm and 58.3% in the TMC group. The n-HA/PA66 group also had better correction of the bisegmental kyphotic angle than the TMC group (7.1° ± 7.5° vs 1.9° ± 8.6°, *p* < 0.01), with lower loss of correction (2.9° ± 2.5° vs 5.2° ± 4.1°, *p* < 0.01). The mean ODI steadily decreased after surgery in both groups. At final follow-up, the ODI and VAS were similar in the TMC and n-HA/PA66 groups.

**Conclusions:**

The n-HA/PA66 cage is associated with excellent radiographic fusion, better maintenance of the height of the fused segment, and better correction of kyphosis than the TMC during 7 years of follow-up after one-level anterior corpectomy. With the added benefit of radiolucency, the n-HA/PA66 cage may be superior to the TMC in anterior reconstruction of thoracic or lumbar fractures.

## Background

For thoracolumbar burst fractures, anterior corpectomy is an important treatment option [1, 2]. Anterior approaches allow greater access to the ventral aspect of the canal without sacrificing the spinous processes, laminae, facets, and intervening ligaments [1–4]. Compared with the posterior approach, the anterior approach requires fixation to only one level above and one level below the fracture to successfully correct kyphosis and maintain this correction over time [3–6].

In the anterior approach, a tricortical autologous bone graft is traditionally applied for spine reconstruction. However, construct stability remains an issue [1]. Problems with allograft bone include graft collapse, graft fracture, nonunion, and donor-site complications [7–10]. Anterior corpectomy with a titanium mesh cage (TMC) is a safe and effective surgical treatment for thoracolumbar fracture, offering better and more predictable results with fewer donor-site complications and earlier biomechanical stabilization than iliac bone grafting [10–13]. However, TMCs have a fairly large amount of subsidence with a large Young’s modulus, which may affect the long-term maintenance of kyphosis [12]. In the last few decades, novel titanium expandable cages and polyetheretherketone (PEEK) cages have been used to reconstruct thoracolumbar burst fractures, with remarkable results reported in terms of stabilization, fusion, and kyphosis correction during short-term follow-up [14–17].

The hollow nano-hydroxyapatite/polyamide66 (n-HA/PA66) cage is a bionic non-metallic cylinder that is easily penetrated by X-rays and CT to evaluate the fusion status [18, 19]. Recently, the n-HA/PA66 cage filled with autograft material has been used for anterior cervical reconstruction, achieving less subsidence and more satisfactory clinical outcomes than the TMC during long-term follow-up [18, 19]. Apart from the lower Young’s modulus, the rims of the n-HA/PA66 cage are wider than the rims of the TMC with lower pressure, which reduces the cutting of the cage into the endplates [19]. Furthermore, the n-HA/PA66 cage reportedly achieves an excellent bone fusion rate during 1 year of follow-up, with no instrument failure [20]. However, few studies have compared the long-term clinical outcomes of the n-HA/PA66 cage and TMC in the reconstruction of thoracic and lumbar fractures.

After the recent wide use of the n-HA/PA66 cage, there is a need to evaluate the long-term subsidence, fusion rate, and kyphosis correction. The purpose of the present study was to compare the long-term outcomes of the TMC and n-HA/PA66 cage in anterior one-level corpectomy and fusion for thoracic and lumbar fractures.

## Methods

This retrospective study included 113 patients with acute traumatic thoracic and lumbar burst fractures (between T10 and L4) who underwent anterior one-level corpectomy and fusion. The inclusion criteria were: (1) The single vertebral burst fractures indicated for surgery were in the T10-L4 vertebrae, with or without other minor vertebral fractures that didn’t require surgery. (2) The fracture type was mainly severe vertebral burst fracture, with or without mild posterior ligament complex injury，including A3, B1 and C1. (3) Patients underwent anterior vertebral body replacement using a titanium or n-HA/PA66 cage between January 2009 and January 2014. (4) Patients aged 18 to 65 who had no previous thoracic or lumbar spine surgery. The exclusion criteria were: (1) Thoracolumbar fractures with severe damage to the anterior and posterior columns of the spine and extremely poor stability，including B2/3 and C2/3. (2) Postoperative clinical and radiographic follow-up period less than 7 years. (3) Pre-existing serious spinal column deformity. (4) Any severe systemic medical disease.

**Fig. 1:**
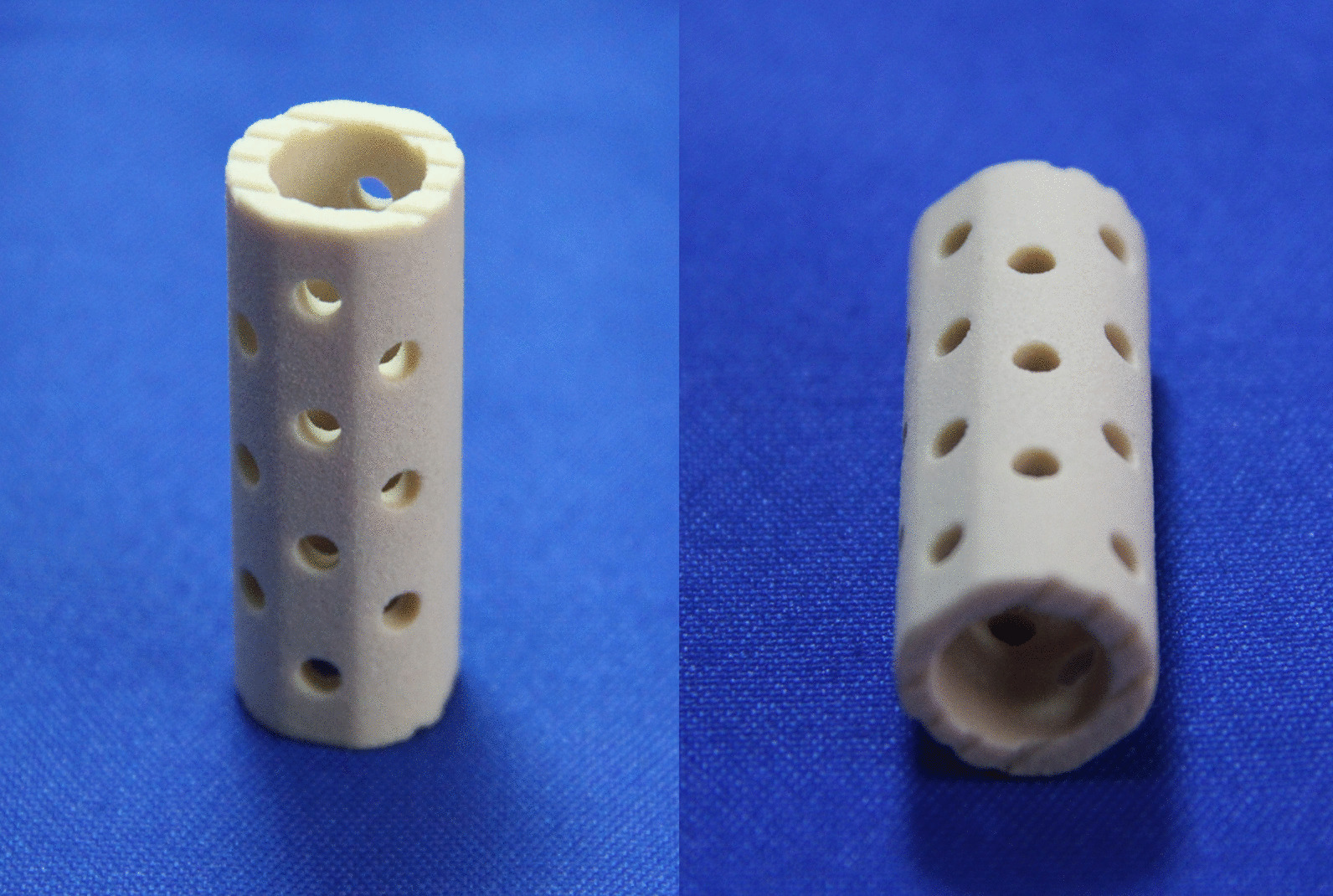
The nano-hydroxyapatite/polyamide-66 cage (Sichuan National Nanotechnology Co, Ltd, Chengdu, China) comes with a variety of commonly used cages

A total of 145 patients underwent surgery during this period; however, 32 patients could not be contacted via telephone calls or multiple mailings. Therefore, the final cohort comprised 53 patients in the n-HA/PA66 cage group and 60 patients in the TMC group. This study was approved by the Ethics Committee of the West China hospital (No. 2019–654). All patients underwent anterior fusion with the n-HA/PA66 cage (Sichuan National Nanotechnology Co, Ltd, Chengdu, China) (Fig. 1) or titanium mesh cage (Medtronic, Memphis, TN, USA) with the anterior segmental instrumentation Z-plate (Medtronic Sofamor-Danek, Memphis, TN) or CD Horizon Antares plate (Medtronic Sofamor-Danek). All surgeries were performed by two attending orthopaedic spine surgeons.

All 113 patients underwent single-level vertebrectomy via an anterior approach (retroperitoneal, transthoracic, or thoracoabdominal) appropriate for the level of the corpectomy. The corpectomy was total or subtotal. The discs above and below the vertebrectomy site were thoroughly resected, and the exposed endplates were decorticated. The appropriate cage was filled with autogenous bone graft material obtained from a morselized vertebral body or harvested rib, and then, the cage was inserted. Anterior stabilization was added from the proximal to the distal vertebrae adjacent to the corpectomy. Finally, the remnant morselized cancellous bone was surrounded anteriorly and laterally by the cage.

All patients had plain radiographs taken preoperatively, postoperatively, and after at least 7 years of follow-up. The interbody height (IH), cage subsidence, and bisegmental kyphotic angle (BKA) were evaluated on lateral plain radiographs. The fused segmental height was defined as the distance between the midpoints of the superior endplate of the cephalic vertebra and the inferior endplate of the caudal vertebra in the fused segment. Height loss of the fused segment was measured as the difference between the immediately postoperative measurements and the follow-up measurements; subsidence was defined as a height loss of more than 3 mm. Cage subsidence of more than 2 mm was defined as radiographic subsidence. The disc height was defined as the average of the anterior and posterior disc heights. The BKA was defined as the angle between the line along the superior endplate of the cephalad adjacent level and the line along the inferior endplate of the caudal adjacent level. We defined the lordosis angle as positive and the kyphosis angle as negative. All radiographic parameters were measured by two attending surgeons who were not involved in the primary surgery, and the average values were used in the analysis.

Three-dimensional CT was performed at 1 year postoperatively and at final follow-up. Two senior surgeons assessed the CT scans to evaluate the fusion status based on the grading system proposed by Brantigan’s grade 5 criteria [21], with grade 4 or 5 showing fusion. The surgery time, blood loss volume, and complications were recorded. The Oswestry Disability Index (ODI) and 10-point visual analogue scale (VAS) were used to assess the clinical outcome and pain, respectively, before surgery, at 3 months after surgery, and at final follow-up.

The statistical evaluation was performed by applying the t-test, chi-squared test and Mann–Whitney U-tests using SPSS 21.0 (SPSS, Chicago, USA). *P* < 0.05 was considered to indicate a statistically significant difference.

## Results

The study cohort comprised 113 patients (68 men, 45 women) who underwent single-level anterior reconstruction of a thoracolumbar fracture with a mean follow-up time of 89 months (83–118 months). An n-HA/PA66 cage was used in 53 patients, while a TMC was used in 60 patients. There were no significant differences between the TMC and n-HA/PA66 groups in sex, age, hospital stay, blood loss volume, or follow-up duration (Table 1).

**Table 1:** Patient demographic data before surgery

Patient demographic data			
Variables	n-HA/PA66 group (53patients)	TMC group (60 patients)	P value
Female gender	22/53	23/60	0.73
Age	43.1 ± 10.4	41.2 ± 11.9	0.37
Operative time(min)	143.4 ± 36.5	151.5 ± 39.7	0.26
Blood loss(ml)	336.5 ± 75.3	351.2 ± 87.5	0.34
Smoker	12/53	16/60	0.62
Follow-up(years)	90.7 ± 11.2	89.2 ± 8.9	0.43
Residual can all (%)	46.3 ± 15.3	48.8 ± 16.2	0.40
AO-spine thoracolumbar spine injury classification			
A	37	42	
B	13	15	
C	3	3	
Injured site			
T12	8	10	
L1	24	29	
L2	11	12	
L3	10	9	
ASIA in admission			0.53
A	2	3	
B	4	5	
C	9	11	
D	20	25	
E	18	17	

*TMC* titanium mesh cage, *n-HA/PA66* nano-hydroxyapatite/polyamide66

**Fig. 2:**
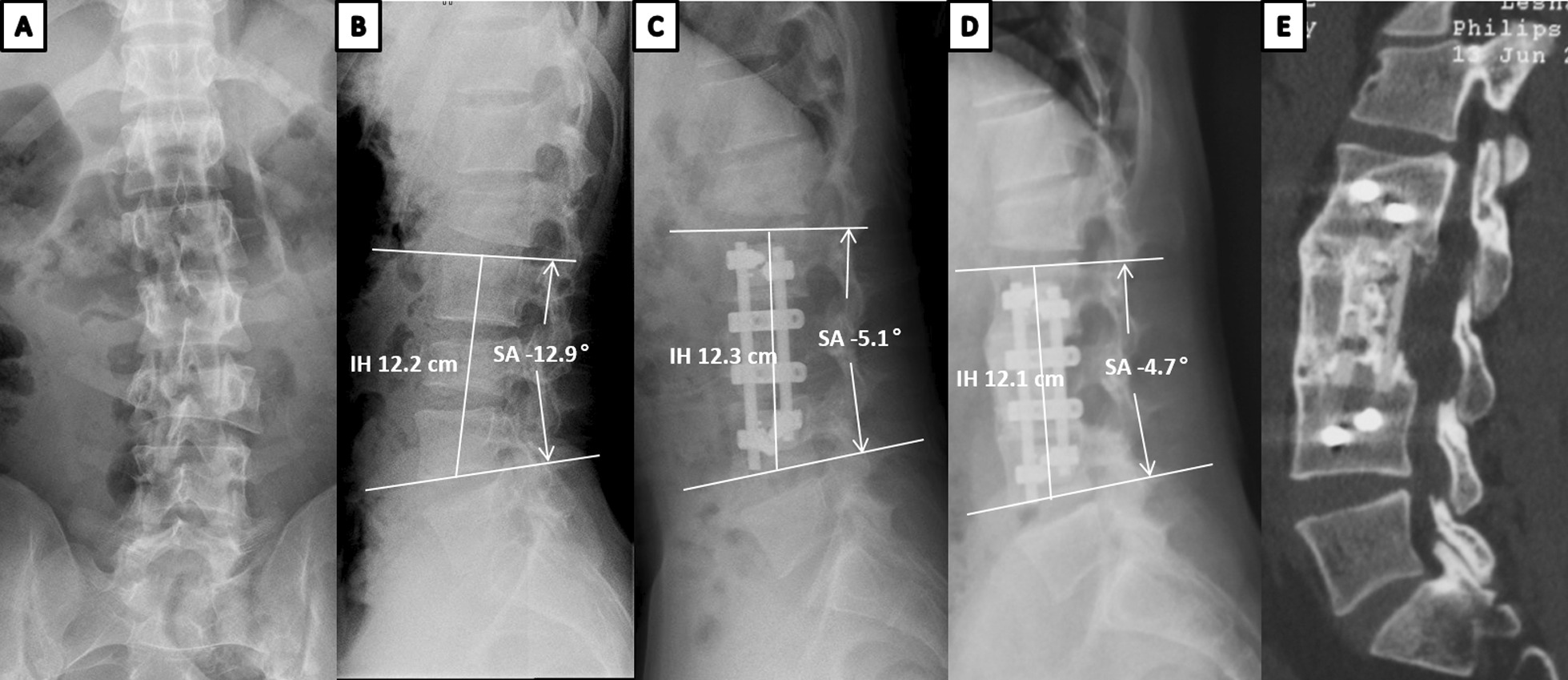
Images obtained in a 30-year-old male patient who underwent anterior short-segment fusion with a n-HA/PA66 cage. The patient had a severe burst fracture of L3 vertebral body, and a mild compression fracture of L1 vertebra which did not require surgery. **A**, **B** Preoperative anteroposterior (**A**) and lateral (**B**) radiographs showing a bisegmental kyphotic angle of − 12.9° and an intervertebral height of 11.9 cm. **C** Postoperative lateral radiographs showing that the BKA was − 5.1° and the IH was 12.3 cm. **D**, **E** Lateral radiograph and sagittal CT images obtained at 7-year follow-up time, showing that the IH changed to 10.9 cm with 2 mm subsidence and the BKA changed to − 4.7°. This patient showed solid fusion at final follow-up time. There is continuous trabecular formation between vertebral bodies and bony bridging outside them

There were no significant differences between the two groups in the preoperative interbody height or BKA. The postoperative and final interbody height did not differ significantly between the TMC and n-HA/PA66 groups (Table 2). However, the loss of interbody height at final follow-up was greater in the TMC group than the n-HA/PA66 group (3.9 mm ± 2.5 mm vs. 2.3 mm ± 1.6 mm, respectively; *p* < 0.01), and the TMC group had a significantly higher incidence of subsidence (58.3% vs. 24.5%, *p* < 0.01). There were no significant differences between the TMC and n-HA/PA66 groups in the BKA preoperatively (− 10.3° vs. − 12.0°; *p* = 0.43), postoperatively (− 2.1° ± 8.0° vs. − 3.2° ± 6.9°; *p* = 0.45), or at final follow-up (− 5.0° ± 9.7° vs. − 8.4° ± 9.9°; *p* = 0.07). The n-HA/PA66 group had a larger final correction of the BKA (7.1° ± 7.5° vs 1.9° ± 8.6°, *p* < 0.01) and lower loss of correction of the BKA (2.9° ± 2.5° vs 5.2° ± 4.1°, *p* < 0.01). Thus, the n-HA/PA66 group had a much lower incidence of subsidence and better correction of the BKA than the TMC group (Fig. 2).

**Table 2:** Radiographic outcomes between the n-HA/PA66 group and TMC group

Radiographic outcomes			
Variables	n-HA/PA66 group (53 patients)	TMC group (60 patients)	P value
Final Brantigan fusion grade			0.64
Grade 5 Fused	45 (84.9%)	49 (81.7%)	
Grade 4 Probable fused	6 (11.3%)	8 (13.3%)	
Grade 3 Uncertain	2 (3.8%)	3 (5.0%)	
Grade 2 Probable unfused	0	0	
Grade 1 Unfused	0	0	
Interbody Height (mm)			
Preoperatively	104.7 ± 9.7	105.0 ± 11.2	0.89
Postoperatively	111.5 ± 8.4	112.4 ± 9.5	0.61
Final follow-up	109.2 ± 8.9	108.5 ± 10.4	0.72
Subsidence (mm)	2.3 ± 1.6	3.9 ± 2.5	< 0.01*
Final subsidence rate	24.5% (13/53)	58.3% (35/60)	< 0.01*
BKA(°)			
Preoperatively	− 12.0 ± 11.7	− 10.3 ± 10.2	0.43
Postoperatively	− 2.1 ± 8.0	− 3.2 ± 6.9	0.45
Final follow-up	− 5.0 ± 9.7	− 8.4 ± 9.9	0.07
Final correction	7.1 ± 7.5	1.9 ± 8.6	< 0.01*
Loss of correction	2.9 ± 2.5	5.2 ± 4.1	< 0.01*

*TMC* Titanium mesh cage

*n-HA/PA66* nano-hydroxyapatite/polyamide66

*BKA* segmental lordosis

^*^p < 0.05

At final follow-up, fusion in the n-HA/PA66 group was classified as grade 5 in 45 patients (84.9%) and grade 4 in six (11.3%), while two patients had asymptomatic grade 3 fusion (3.8%). In the TMC group, fusion was classified as grade 5 in 49 patients (81.7%) and grade 4 in eight (11.3%), while three patients had asymptomatic grade 3 fusion (5.0%). Bony fusion was achieved in 95% (57/60) of patients in the TMC group and 96.3% (51/53) of patients in the n-HA/PA66 group. Some patients in the n-HA/PA66 group showed better fusion at final follow-up than at the 1-year follow-up, with bone growth to the outside surfaces of the n-HA/PA66 cage; this was rarely seen in the TMC group (Table 3).

**Table 3:** Clinical outcomes between the n-HA/PA66 group and TMC group

Clinical outcomes			
Variables	n-HA/PA66 group (53 patients)	TMC group (60 patients)	P value
VAS scale			
Preoperatively	7.3 ± 1.6	6.9 ± 1.8	0.25
3-month postoperative	3.4 ± 1.2*	3.2 ± 1.0*	0.39
Final follow-up	2.2 ± 1.0*^#^	2.1 ± 0.9*^#^	0.42
ODI scores (%)			
Preoperatively	78.1 ± 22.3	80.7 ± 24.1	0.55
3-month postoperative	37.9 ± 16.8*	36.8 ± 18.5*	0.73
Final follow-up	17.8 ± 11.9*^#^	19.2 ± 13.4*^#^	0.54

*TMC* Titanium mesh cage; *n-HA/PA66* Nano-hydroxyapatite/polyamide66, *VAS* 10-point visual analogue scale, *ODI* Oswestry disability index score

^#^*p* < 0.05 compared with 3-month post-operation

^*^p < 0.05 compared with preoperation

The preoperative ODI and VAS were not significantly different between the n-HA/PA66 and TMC groups. The VAS improved in both groups during follow-up and did not significantly differ between the n-HA/PA66 and TMC groups at final follow-up. The postoperative ODI was similar in the TMC and n-HA/PA66 groups. The ODI also improved in both groups during follow-up. However, the mean ODI in the n-HA/PA66 group at 7 years postoperatively was still similar to the mean ODI in TMC group.

**Fig. 3:**
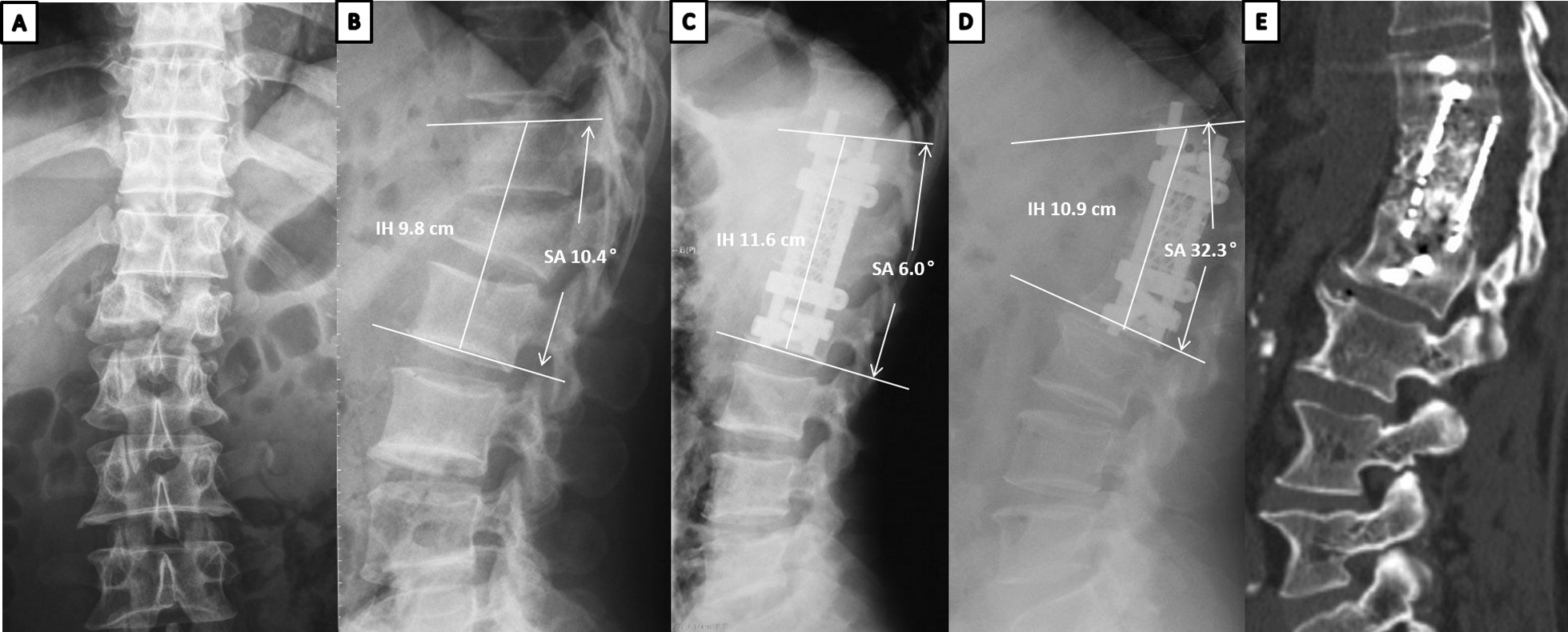
Case example showing a 48-year-old female patient who underwent anterior short-segment fusion with a TMC cage after anterior one-level corpectomy at L1. **A**, **B** Preoperative anteroposterior (**A**) and lateral (**B**) radiographs showing a bisegmental kyphotic angle of 10.4° and an intervertebral height of 9.8 cm. **C** Postoperative lateral radiographs showing improvement of the BKA to 6.0° and IH improvement to 11.6 cm. **D**, **E** Lateral radiograph and sagittal CT images obtained at 8-year follow-up time, showing severe subsidence with screw broken. The IH reduced to 10.9 cm with 7 mm subsidence and the BKA increased to 32.3°

There was no cage migration or breakage in either group at final follow-up, and no revision surgery was required due to cage-related complications. Two patients in the TMC group had broken screws related to severe cage subsidence. Both groups had one patient with crew loosening. However, no patients had severe wound infection after surgery. The patients who did not exhibit bony fusion at final follow-up did not undergo revision surgery because they had no discomfort.(Fig. 3).

## Discussion

Severe thoracolumbar fractures often cause great damage to the spinal structure and often require 360° fixation to rebuild spinal stability. However, for the single vertebral burst fractures with or without mild posterior ligament complex injury, good spinal stability can also be reestablished through anterior approach alone [10, 20, 22, 23]. A dual-rod cross-connector construct is significantly more rigid than a single-rod construct and could be more suitable for these patients with thoracolumbar fractures [23]. In this process, the selection of support materials is particularly important. The n-HA/PA66 cage has been used as a bionic non-metallic material in reconstructive operations of spine in the past decade. HA has good osteoconductivity and has been well accepted as a bone repair substitute, while PA66 is a polymer with strong intensity, high flexibility, and good stability [24–26]. Furthermore, the n-HA/PA66 composite exhibits excellent biocompatibility and osteogenesis in vivo [27] and is an ideal microstructure material with a dynamic perfusion culture condition that improves osteogenesis [28]. In the present study, the n-HA/PA66 cage showed great bionic ability to achieve osteogenesis and osseointegration in the long-term (bone fusion rate of 96.2%). Bone grew to the outside surfaces of the n-HA/PA66 cage, with cortical bone and newly formed bone tracked along the inside surface. In contrast, the non-bionic surface of the PEEK cage often presents as the typical non-reactive fibrous tissue interface [29, 30], resulting in inferior osseointegration [30]. A previous study reported that 20% of PEEK cages exhibit impaction on the upper plateau during a minimum follow-up of 8 months [16].

As a bioactive material with the ability to promote new bone formation and provide a scaffold for osteogenesis, the n-HA/PA66 strut has advantages in the anterior reconstruction of thoracic and lumbar corpectomy. Ou et al. [31] reported that the n-HA/PA66 strut achieved a satisfactory short-term clinical outcome, with an excellent fusion rate of nearly 100%. Yang et al. [20] also reported that the n-HA/PA66 cage achieved a low subsidence rate of 19.6% and great fusion rate of 90.2% during 2 years of follow-up. The elastic modulus of the n-HA/PA66 strut is 5.6 GPa, which is similar to the elastic modulus of natural bone [18–20] and much lower than that of the TMC (110 GPa). Furthermore, the n-HA/PA66 strut avoids some of the stress shielding caused by metallic implants and promotes bony fusion. The use of the TMC in anterior column reconstruction of the thoracolumbar spine often results in severe subsidence [12, 13, 32]. Dvorak [12] reported an average TMC subsidence of 4 mm, but with acceptable correction of vertebral kyphosis at final follow-up. Jang et al. [32] found that TMC subsidence occurred in 93.3% of patients after anterior cervical corpectomy and reconstruction. In the present study, the TMC group had a subsidence rate of 58.3% during 7 years of follow-up.

The long-term subsidence rate in the n-HA/PA66 group in our study was 24.5%, with a mean subsidence of 2.3 mm; this was higher than the subsidence rate reported in a previous study [20] and higher than the subsidence rate of nearly 20% reported in the cervical spine [19]. The elastic modulus of the cartilage endplate and cancellous bone (0.1–0.5 GPa) is lower than the elastic modulus of the n-HA/PA66 cage, causing posterior subsidence at the interface with the cancellous bone. The relatively high subsidence rate in the n-HA/PA66 group may be due to the difficulty in the shaping of the n-HA/PA66 cage during surgery, which makes it harder to match both the superior and inferior endplates. Large mismatched angles are an important factor leading to increased cage subsidence [33, 34].

Owing to the greater loss of height of the fusion segments, severe subsidence was correlated with loss of BKA correction and subsidence-related complications. Deml reported 10.5° correction of kyphosis and 1.6° loss of correction using the PEEK cage in anterior reconstruction after thoracolumbar corpectomy through the anterior–posterior approach [17]. In surgery via the anterior approach, many previous studies have showed a mild loss of correction using a PEEK cage or TMC with short-term follow-up [10, 13, 35]. However, with significant subsidence of the TMC, the correction of kyphosis can no longer be kept stable [12]. Brandao et al. [16] also found no significant difference in the loss of correction between a TMC and a PEEK expandable cage, despite much higher loss of correction in both groups (8.86° vs. 3.65°). In our study, the loss of correction was 5.2° in the TMC group, which was similar to the 4.2° reported in a previous study [12]. In addition, the n-HA/PA 66 cage showed significantly better correction of the BKA and lower loss of correction than the TMC group after 7 years of follow-up than the TMC, which might be due to the lower elastic modulus and better osteoconductivity of the n-HA/PA 66 cage.

There were no significant differences in the ODI and VAS between the n-HA/PA66 and TMC groups at final follow-up [36, 37]. In addition, the n-HA/PA66 cage exhibited excellent biocompatibility and osteoconductive ability [27, 38, 39]. Considering the lower elastic modulus with more stable correction of kyphosis and earlier bony fusion of the n-HA/PA66 group compared with the TMC group, the n-HA/PA66 seems to be an ideal cage to replace the TMC in anterior reconstruction of thoracolumbar fractures.

The present study has some limitations. The sample size was small, the choice of the cage was not randomized, and the results may have been influenced by physician factors to a certain degree. A future multicentre study with a larger sample size is warranted to compare the long-term effects of these two cages.

## Conclusion

The n-HA/PA66 cage is associated with lower subsidence and better correction of kyphosis than the TMC at 7 years after one-level anterior reconstruction of thoracolumbar fracture. With the added benefits of radiolucency, a lower elastic modulus, and better osteoconductivity, the n-HA/PA66 cage may be better than the TMC in anterior thoracolumbar construction.

## Data Availability

Data will be available upon request to the first author Bowen Hu.

## Abbreviations

TMC: Titanium mesh cage
PEEK: Polyetheretherketone
n-HA/PA66: Nano-hydroxyapatite/polyamide66
BKA: Bisegmental kyphotic angle
IH: Interbody height
ODI: Oswestry disability index
VAS: 10-Point visual analogue scale
CT: Computed tomography
